# Measuring What Matters: Days Alive and Out of Hospital and Health Care Utilization in Kidney Transplantation

**DOI:** 10.34067/KID.0000001081

**Published:** 2026-03-26

**Authors:** Joel T. Adler

**Affiliations:** Division of Transplantation, Department of Surgery and Perioperative Care, Dell Medical School, The University of Texas at Austin, Austin, Texas

**Keywords:** economic analysis, kidney transplantation, patient-centered care, quality of life

Transplantation is both a high-risk and high-utilizing therapy. Because organs are scarce, clinicians and systems continually balance risks and benefits—not only for individual candidates but also for centers and payers. Although candidacy decisions are rarely made solely on anticipated post-transplant health care use, it is hard to imagine that expected health care utilization plays no role in clinical judgment—and surprising how little we actually know about post-transplant health care utilization in high-need populations such as older adults.

Punukollu *et al*.^1^ contribute by foregrounding patient-facing utilization metrics in elderly kidney recipients. In a single-center cohort of 613 deceased donor transplants over a decade, they compared outpatient and inpatient utilization between recipients of lower versus higher Kidney Donor Profile Index (KDPI) kidneys. As expected, lower KDPI grafts had better function and lower death-censored graft loss.^2^ More importantly for many patients, the authors report similar days alive and out of hospital (DAOH) at 90 and 365 days across KDPI strata—a reflection of the quality-of-life benefits that kidney transplantation can offer.

DAOH is useful because it synthesizes survival and health care exposure into a single, patient-salient metric—essentially, time living life at home rather than in facilities^3^—an outcome that aligns especially well with older adults' priorities.^4^ Its main weakness is specification sensitivity: DAOH can be distorted by conditioning on survival (*e.g*., calculating DAOH-365 only among survivors to day 365) and by incomplete capture of postacute or out-of-network days that may not be easily captured by investigators; likewise, summary choice and rounding (mean versus median/interquartile range) can also mask tails of the distribution. The distribution of DAOH matters: means and medians can hide outliers, and, as well documented in US data, a small tail of patients account for a disproportionate share of health care use.^5^

The study's utilization findings will reassure some readers that accepting higher KDPI organs need not entail a dramatically greater post-transplant care burden—at least within a center that routinely preconsents older candidates for high KDPI. At the same time, observational comparisons in elderly cohorts are sensitive to selection bias (even with statistical methods) and outcome construction. Factors that are hard to measure (frailty, cognition, and social support) shape who receives which offers and how they fare^6^; consequently, observed similarities may understate or overstate true differences.

Crucially, this study does not address the decision older adults actually face: accept a higher-KDPI kidney now or wait for a lower KDPI offer. For many elderly patients, time on dialysis drives morbidity, mortality, and quality-of-life losses; earlier transplantation—even with a higher KDPI organ—can dominate waiting for perfect. Framing the choice as fast versus durable kidney, rather than good versus bad kidney, better reflects the trade-off older patients confront and what many value: days alive and at home.

Practical steps exist to reduce avoidable post-transplant utilization without restricting access: standardize early follow-up bundles (medication reconciliation+laboratory results), add brief nurse-led phone/telehealth check-ins, strengthen adherence/immunosuppression management (*e.g*., pharmacist- or mobile-health–supported), and target supports for frailty and caregiver needs—approaches associated with fewer adverse events or readmissions in transplant patients.^7^ Telehealth, in particular, can offload travel and enable rapid issue triage for stable recipients; as virtual care expands in nephrology^8^ and transplantation,^7,9^ clear protocols and risk-adjusted evaluation will help ensure access gains translate into lower avoidable utilization rather than selection effects.

Methodologically, future work in this space should (*1*) report DAOH transparently (window definition, survival conditioning, and out of network capture) with distributions; (*2*) use recurrent-event models for readmissions/visits and competing-risk frameworks for graft loss and mortality in elderly recipients; and (*3*) emulate the clinical decision with target-trial designs contrasting accept high KDPI now versus decline and wait. Subgroup and equity-focused analyses (*e.g*., by rurality, dual eligibility for Medicare and Medicaid) can guard against policies that inadvertently penalize vulnerable patients.^10^

What we measure should reflect what matters. DAOH, health care utilization, and related end points are useful precisely because they align with what many older adults prioritize—time alive at home and functional independence—but they must be built and reported transparently. Studies like these help us provide realistic expectations and better post-transplant care for older recipients.
